# Dynamic Modeling of Silicon Bioavailability, Uptake, Transport, and Accumulation: Applicability in Improving the Nutritional Quality of Tomato

**DOI:** 10.3389/fpls.2018.00647

**Published:** 2018-05-17

**Authors:** Mari C. López-Pérez, Fabián Pérez-Labrada, Lino J. Ramírez-Pérez, Antonio Juárez-Maldonado, América B. Morales-Díaz, Susana González-Morales, Luis R. García-Dávila, Jesús García-Mata, Adalberto Benavides-Mendoza

**Affiliations:** ^1^Departamento de Horticultura, Universidad Autónoma Agraria Antonio Narro, Saltillo, Mexico; ^2^Departamento de Botánica, Universidad Autónoma Agraria Antonio Narro, Saltillo, Mexico; ^3^Robótica y Manufactura Avanzada, Centro de Investigación y de Estudios Avanzados Unidad Saltillo, Ramos Arizpe, Mexico; ^4^Departamento de Horticultura, CONACYT-Universidad Autónoma Agraria Antonio Narro, Saltillo, Mexico; ^5^Cosmocel España, Zaragoza, Spain; ^6^Cosmocel Brasil, São Paulo, Brazil

**Keywords:** silicates, nutritional quality, stress tolerance, mathematical models, silicon and health

## Abstract

Silicon is an essential nutrient for humans, additionally is beneficial for terrestrial plants. In plants Si enhances tolerance to different types of stress; in humans, it improves the metabolism and increases the strength of skeletal and connective tissues as well as of the immune system. Most of the Si intake of humans come from edible plants creating a double benefit: first, because the absorption of Si increases the antioxidants and other phytochemicals in plants, thereby increasing its functional value, and second because the higher concentration of Si in plants increases intake in human consumers. Therefore, it is desirable to raise the availability of Si in the human diet through the agronomic management of Si accumulator species, such as corn, wheat, rice, soybeans, and beans. But also in such species as tomatoes, carrots, and other vegetables, whose per capita consumption has increased. However, there are few systematized recommendations for the application and management of Si fertilizers based on the physicochemical factors that determine their availability, absorption, transport, and deposition in cells and tissues. This study presents updated information about edaphic and plant factors, which determine the absorption, transport, and deposition rates in edible organs. The information was integrated into an estimated dynamic model that approximates the processes previously mentioned in a model that represents a tomato crop in soil and soilless conditions. In the model, on the other hand, was integrated the available information about key environmental factors related to Si absorption and mobilization, such as the temperature, pH, and soil organic matter. The output data of the model were compared against information collected in the literature, finding an adequate adjustment. The use of the model for educational or technical purposes, including the possibility of extending it to other horticultural crops, can increase the understanding of the agronomic management of Si in plants.

## Introduction

On average a human organism contains 1–2 g of Si, being the third most abundant trace element after Fe and Zn. When it is contained in food in adequate quantity, silicon is effectively absorbed by the human organism (Sripanyakorn et al., 2009), transferring to practically all tissues, but concentrating in greater quantity in the connective tissues (O'Dell and Sunde, 1997; Jugdaohsingh, 2007). With a diet rich in vegetables the daily intake of silicon is between 140 and 204 mg Si day^−1^; however, in western populations with lower consumption of vegetables, the daily intake can range between 20 and 50 mg day^−1^. Silicon is rarely toxic when taken orally (Arora and Arora, 2017), with a recommended maximum intake of 1,500 mg day^−1^ (White and Broadley, 2005). On the other hand, the minimum value of Si consumption to achieve some benefits has been determined at 25 mg day^−1^ (Nielsen, 2014). After ingestion, most of the absorbed Si is excreted in the urine (Jugdaohsingh, 2007), most likely as orthosilicic acid and/or magnesium orthosilicate.

Plant foods are the primary source of Si in the human diet. This includes grains of cereals (rice, wheat, oats, and barley) and less refined products of cereals, fruits (bananas and apples), vegetables (potato, beet, carrot, bean, spinach, and lentils) (Powell et al., 2005), and beverages such as beer since the Si contained in barley and hops is solubilized during the manufacturing process (Pennington, 1991; Powell et al., 2005; Jugdaohsingh, 2007). Other sources of silicon are meat, fish, milk, and eggs (Nielsen, 1974; Nuurtamo et al., 1980). Drinking water can also be a source of Si depending on the source and the method of processing (Jugdaohsingh, 2007; Sripanyakorn et al., 2009).

In plants Si is not considered an essential element, but it has been found that its inclusion in fertilizer formulations provides higher tolerance to stress (Adrees et al., 2015; Rizwan et al., 2015; Cooke and Leishman, 2016; Luyckx et al., 2017), especially on soilless growing conditions (Epstein, 1994, 2009; Voogt and Sonneveld, 2001). An additional benefit of adding silicon in the fertilization of crops is related to the more significant amount of silicon available to human consumers. In other words, the use of silicon in agricultural production brings a benefit to agricultural producers in the form of stronger and stress-tolerant plants, while for consumers of harvested products it gives an advantage in the way of higher silicon intake in the food.

The use of mathematical models in the mineral nutrition of plants allows to simulate the dynamics of the absorption of water and dissolved ions in response to different internal and external factors (Juárez-Maldonado et al., 2017). The models contribute to the quantitative understanding of the factors involved in the absorption, transport, and accumulation of mineral elements; additionally, they allow to explore different environmental or endogenous situations that modify the nutrition of the plant (Mankin and Fynn, 1996). Regarding Si modeling, (Sakurai et al., 2017) presented a dynamic model of Si absorption and transport for rice. The model was integrated considering the activity of different transporters and the distribution of Si through different nodes in the entire plant; the model was able to predict the dynamic behavior of silicon in the plant successfully. However, in the case of vegetables, there are no models that consider silicon, although there are published models that effectively simulate nutrition with other mineral elements (Juárez-Maldonado et al., 2014b, 2017; Ramírez-Pérez et al., 2018).

The aim of this manuscript was the integration of an estimated dynamic model that approximates the availability, absorption, transport, and accumulation of silicon in a tomato crop in soil and soilless conditions.

## Benefits of silicon in horticultural plants

The ferns, horsetails, and grasses such as corn, wheat and rice, and sugar cane, are the plants that naturally accumulate more silicon (Liang et al., 2015). However, in the presence of adequate amounts of silicon in the form of Si(OH)_4_, all plants, including horticultural species such as tomato and cucumber, absorb it. Plants use silicon in a manner not yet well understood to stimulate the antioxidant metabolism, the processes of plant's hardening, defense, and adaptation to environmental factors. Depending on whether they are species that carry out silicification, Si(OH)_4_ is concentrated in polymeric form (amorphous hydrated silica) in different cellular and extracellular compartments, finally transforming it using a deposition process dependent on transpiration into insoluble biogenic silica (SiO_2_.nH_2_O) which forms structures called phytoliths or opal (Sangster et al., 2001; Katz, 2014; Exley, 2015). The biogenic silica is subsequently incorporated into the soil contributing with 1–3% of the total Si in the soil (Desplanques et al., 2006).

The silicon absorbed by the plants seems to be maintained under constant exchange between the soluble forms (Si(OH)_4_) and the insoluble fraction (polymeric silicic acid and biogenic silica) (Exley, 2015). Most of the Si deposited as biogenic silica remains as such throughout the life of the plant (Sangster et al., 2001). The soluble part is directly available to humans when they consume plant foods, while the insoluble fraction could perhaps be considered as an integral part of the fiber. The environmental factors and differences between plant species that modify the ratio Si soluble/Si insoluble, which ultimately determines the dietary utility of the product, have not been studied.

Considering that, (i) all plants seem to have the capacity to absorb silicon (Exley, 2015), (ii) and in view of the rise that has taken in recent years the production of vegetables using soilless production systems (Pignata et al., 2017), (iii) in addition to the fact that irrigation water and horticultural substrates provide little bioavailable silicon (Liang et al., 2015), then, it would be advisable to include silicon on a daily basis in fertilizer formulations used in those soils with low Si bioavailability as well as in soilless crops for the production of vegetables under protected conditions (Epstein, 1994, 2001).

The different groups of plants have different capacity to mobilize Si toward their various organs, but practically all absorb the silicon from the soil when it is available in the soil solution or the nutrient solution. The species with low mobilization capacity accumulate it in the roots and stems, while the species with high mobilization capacity accumulate it in stems, leaves, fruits, and seeds. Si appears to be absorbed in the form of Si(OH)_4_ by channels belonging to the aquaporins' group. Thus the rate of absorption and transport depends on the flow of water linked to transpiration (Exley, 2015; Sakurai et al., 2017).

The cereals are plants with a high capacity of silicification and therefore represent a significant amount of Si in the diet. However, the silicon contained in cereals will be encountered almost all in the form of insoluble biogenic silica (Sangster et al., 2001) which would be partially dissolved by the acids of the digestive system; on the other hand, in fruits and vegetables, due to their lower silicification capacity, it is expected that there will be more soluble silicon, which theoretically would be more available to be assimilated during intake. Considering the above, it is possible that the fruits of horticultural species such as tomato can be excellent sources of Si for the diet.

Additionally, it is known that in comparison with the dicotyledons, cereals contribute less Ca and Mg (White and Broadley, 2005). Therefore, a diet high in cereals that provides a significant amount of Si on average will contain less Ca and Mg than a mixed diet with base in cereals and dicots. On the other hand, the consumption of vegetables and fruits has grown considerably in recent decades among the human population and it is desirable that species such as tomatoes, eggplants, strawberries, cucumbers, avocados, melons, watermelons, carrots, onions, chilies, pumpkins, among others, contain a higher amount of silicon, considering the double benefit already mentioned of the crop higher tolerance to stress and the contribution of Si to human consumers.

In soilless crops, it is necessary to consider the contribution of Si in fertilizers since irrigation water does not provide enough, only from 5 to 24 mg L^−1^ Si (Liang et al., 2015). The lowest recommended concentration of Si in the nutrient solution for plants growing on substrates other than soil is 28 mg L^−1^ Si (Epstein, 1994), which can be achieved with 123 mg L^−1^ of Na_2_SiO_3_.

## Edaphic factors that determine the availability of silicon

Si is found in soil as an inert mineral in the form of quartz or aluminosilicates such as micas and feldspars. The weathering of these materials by rainwater, irrigation water, or by the acid metabolites of microorganisms and plant roots produce Si(OH)_4_ that under a balanced condition reaches a concentration of up to 1.8 mM (173 mg L^−1^, equivalent to 50.4 mg L^−1^ Si). Above this level, reaching 2 mM (192.18 mg L^−1^), Si(OH)_4_ forms hydrated amorphous silica polymers containing Si unavailable for plants (Epstein, 2001; Liang et al., 2015).

The actual value of the concentration of Si(OH)_4_ in the soil solution is much lower than 1.8 mM, commonly found between 0.1 and 0.6 mM (9.61–57.66 mg L^−1^), but with such low values as 0.02 mM (1.92 mg L^−1^) in very eroded soils (Epstein, 2001; Liang et al., 2015).

The concentration of bioavailable Si in soil solution results from the release rate of Si(OH)_4_. Bioavailability is dependent on the silicon content of the soil minerals, organic matter, the temperature, the amount of precipitation and the acidity of the soil or soil pore water. The incorporation of Si in plants occurs at a rate dependent on the intensity of the transpiration (Exley, 2015), so that conditions of rapid growth can rapidly decrease the availability of Si in the soil solution (Epstein, 2001; Liang et al., 2015).

Soils of tropical areas where high precipitation occurs as well as calcareous and sandy soils of semi-arid and arid regions with low vegetation provide low quantities of Si to the soil solution, so it is recommended to use fertilizers with Si (Epstein, 1999). An affordable source of Si is siliceous sand that is offered in different granulometries, and that is used in quantities of between 500 and 4,000 kg ha^−1^. On the other hand, Mollisol and Vertisol soils of the temperate and subtropical regions are soils that can provide an adequate amount of silicon (Epstein, 2001; Gérard et al., 2008). However, this has not been corroborated in regards of the actual availability of Si(OH)_4_ in soil pore water, since there is little-published information about concentrations, dynamic behavior, and association with other edaphic components of Si in the solution of the soil.

The temperature exerts a substantial impact on the soil solubilization rate of Si. However, the seasonal changes in temperature are significant as a determinant of the Si concentration in the soil solution only in the cold seasons of temperate zones, because the range of temperatures suitable for the growth of a crop is also adequate for the solubilization reactions of silicon in the soil (Sommer et al., 2006). Therefore, the temperature is not considered as a factor subject to management regarding the bioavailability of Si for crop plants. Possible exceptions would be crops in soil mulching and crops grown in greenhouse soil or tunnels. In both cases, soil or substrate temperatures are more stable, and on average higher than those of uncovered soil, in addition to water management more precise in time and quantity, so the bioavailability of Si is expected to be higher.

Another factor regulating the availability of Si(OH)_4_ is the pH of the soil pore water, that depends on the pH of the rainwater or irrigation water and is also modified by the respiratory activity and extrusion of organic acids by microorganisms and plant roots (Pérez-Labrada et al., 2016). In fact, the presence of Si induces the synthesis of citric acid in plants (Hernandez-Apaolaza, 2014). The pK1 of Si(OH)_4_ is 9.6, which indicates that its bioavailability in a nutrient solution is practically unaltered with pH values lower than 9. In the study of Gérard et al. (2008), there was little impact of pH on the bioavailability of Si in the soil solution, but the study conditions were developed under a very narrow range of pH variation. It will be necessary to collect data in different types of soil, or in soils subjected to treatments that modify its reaction or the pH of the soil pore water, to determine the effect of pH on the concentration of Si(OH)_4_.

Both a nutrient solution and the soil solution contain components that modify pH and interact with Si. With a pH> 7 that promotes the formation of Fe hydroxides, an adsorption process occurs that causes the polymerization of Si(OH)_4_. With pH < 6 Si(OH)_4_ begins to polymerize on surfaces with minerals containing Fe, while Al^3+^ would promote the stabilization of Si(OH)_4_ polymers, which would make Si unavailable for plants (Sommer et al., 2006). Considering this, it is possible that the availability of Si in the soil solution is higher with pH values between 6.0 and possibly 7.5 (maybe showing some resemblance to the pattern of bioavailability of P), as long as the soil parent material provides Si in sufficient quantity. Calcareous soils, which naturally have pH values> 8 in the soil solution (Pérez-Labrada et al., 2016), do not provide enough Si (Liang et al., 1994). Thus the fertilizer contributions with Si in crops in calcareous soils are beneficial (Zhang et al., 2017).

Another factor to consider regarding the availability of Si(OH)_4_ in the soil pore water is soil organic matter (SOM) and its dissolved forms. SOM have a profound impact on the availability of mineral elements (Diacono and Montemurro, 2010), either directly by chemical processes or indirectly by the promotion of bacteria and fungi that solubilize Si and other elements of soil minerals (Landeweert et al., 2001). An expected effect of SOM would be the adsorption of Al^3+^ through organic acids (Rustad and Cronan, 1995), which would decrease the Al-Si association and increase the concentration of Si(OH)_4_ available in the soil solution. The organic acids derived from SOM are also agents that promote dissolution in mineral surfaces (Drever and Stillings, 1997) so that in soils with silicon-rich parent materials or agricultural soils with the application of Si fertilizers would be very helpful. In nutrient solutions for soilless crops, the use of organic acids can also be useful to improve the solubility of fertilizers with silicon. On the other hand, indirect evidence is available that indicates that SOM is a factor that increases the bioavailability of Si for crop plants (Ding et al., 2008; Sun et al., 2017), thereby SOM management should be considered to increase the availability of Si for crops.

## The model

The information in the previous section highlighted the factors that can be subjected to management in a crop, both in soil and soilless, with the purpose of increasing the availability of Si(OH)_4_ for plants. In the crops grown in soil, a primary factor is the silicon content of soil's parent material. In the fertile soils of temperate and subtropical zones, Si inputs are rarely required in the fertilizers since the soil will undoubtedly provide the necessary amount. On the other hand, in the calcareous soils of arid and semi-arid regions, and in the soils of tropical regions subject to regimes of intense precipitation, the application of Si with fertilizers will be necessary, but also the consideration of the pH and organic matter management of the soil to ensure adequate availability of Si(OH)_4_.

In soilless crops, the critical factor to consider will be the concentration of Si in the irrigation water. Values below 28 mg L^−1^ Si point out the need to provide Si up to a maximum of 50 mg L^−1^. The management of pH is the next factor to be considered. However, the data presented indicate that pH management aimed at ensuring the bioavailability of P in nutritive solution (that is, maintaining it between 5.5 and 7) will be adequate.

A Matlab-Simulink model (the archives are included in Supplementary Material) is presented below which allows verifying the impact of different environmental scenarios, both in a soil crop and in a soilless crop, using as a model tomato plants. There is also an example of the use of the software to obtain the estimated impact of the environmental variables on the absorption of Si by the tomato plants. The data presented in the previous parts of the manuscript can be tested in this model by verifying the result regarding the concentration of Si in the plants. The purposes of the use of the model are educational or technical, and from our perspective, the model can be useful in the agronomic management of Si in a tomato crop and, possibly applicable to other horticultural crops.

### Description of the model

Tomato (*Solanum lycopersicum* L.) was used as a biological model to describe the distribution of silicon accumulation in the different organs. To describe the effects of the various environmental factors mentioned, the deterministic mathematical model proposed by Tap (2000) and modified by Juárez-Maldonado et al. (2014b) will be used as a basis.

The model consists of six state equations, using as inputs the radiation (PAR, μmol m^−2^ s^−1^), temperature (°C), and CO_2_ concentration (μL L^−1^). The model allows to directly considering the effect of these three variables on the accumulation of silicon in the different organs of the tomato plant.

The scope of the model refers to environmental conditions where intense stress does not prevail since it is assumed that the growth rate of the plants will be a direct function of the irradiance and temperature.

Within the plant, silicon accumulates in different organs depending on the corresponding transpiration rates. Thus, it is necessary to calculate the transpiration by a tomato plant dynamically. For this, the equation 1 is used, based on the fact that a linear correlation can be considered between the biomass accumulated by the tomato plant and its transpiration (Juárez-Maldonado et al., 2014a).

(1) $$\begin{array}{r} Transpiration=Biomass*plm/tcg \end{array}$$

*Biomass* is the mass of the tomato plant in g m^−2^; *plm* is a parameter of the linear model (8.5714); and *tcg* is the time of crop growth (10279801 *s*).

Assuming that the tomato plant does not present a substantial accumulation of Si (Liang et al., 2015), the maximum absorption limit was set for the model at 1% (as SiO_2_) of the dry biomass (Miyake and Takahashi, 1978). In this condition, and while there is an unlimited availability of silicon in the soil solution, the accumulation of maximum total silicon (MSiT) in the plant would be as follows:

(2) $$\begin{array}{r} \text{MSiT}=\frac{Biomass}{100}*PD \end{array}$$

Where *PD* is the planting density expressed in plants m^−2^, which for this model was established in 3 plants m^−2^. This plant density was used by Juárez-Maldonado et al. (2014b) and provide the best financial margin, high yield, and fruit quality (Peet and Welles, 2005).

The distribution of accumulated silicon in the tomato plant will then follow the different transpiration rates of its organs, that is, leaves> stem> fruits ≥ root. In the particular case of tomato, organ transpiration can be approximated to the following percentages of total transpiration: leaves = 90%, stem = 5%, fruits = 2.5%, and root = 2.5%.

Even though the potential availability of Si(OH)_4_ in the soil solution is 192.18 mg L^−1^ (Epstein, 1999; Liang et al., 2015), disponibility is affected by temperature, pH, and organic matter content of the soil. In addition to the factors that are modified with agricultural management such as soil moisture and soil profile.

According to the literature, the availability of silicon in soils is directly affected by soil temperature (Epstein, 1999; Liang et al., 2015). Although there is no clear explanation of how this behavior occurs, it is possible to approach it with a third-order model (Equation 3). This is due to the disponibility of silicon is between 8 and 35°C, being its highest availability at 25° C.

(3) $$\begin{array}{r} T3*Temp^{3}+T2*Temp^{2}+T1*Temp+T0 \end{array}$$

Where *T*3, *T*2, *T*1, *and T*0 are the parameters of the third-order model (equivalent to −0.0003; 0.0127; −0.1093; and 0.1674 respectively), and *Temp* is the 0–30 cm soil temperature (°C).

Concerning the SOM, it is known that there is a positive correlation with the availability of silicon (Ding et al., 2008; Sun et al., 2017). View from an agricultural perspective, soil is rich in organic matter when it has a concentration of 5%. An adjustment with a Michaelis-Menten function was used to describe the higher availability of Si, due to the effect of SOM. For this, the following Equation (4) was used.

(4) $$\begin{array}{r} Vmax*\frac{OM}{\left(Km+OM\right)} \end{array}$$

Where *Vmax* is the parameter of maximum availability of silicon due to organic matter normalized to 1 (*Vmax* = 1). *OM* is the amount of organic matter contained in the soil (%, w/w). And *Km* is the Michaelis-Menten parameter (Km = 2.5).

The pH is also a determining factor in the availability of silicon (Liang et al., 2015). This factor, as well as temperature, is related to the availability of silicon that can be approached to a third-order model. The availability of silicon in soil occurs in the pH range from 2 to 9, with a pH of 7 being the highest availability. Therefore, its effect can be described as follows:

(5) $$\begin{array}{r} pH3*pH^{3}+pH2*pH^{2}+pH1*pH+pH0 \end{array}$$

Where *pH*3, *pH*2, *pH*1, *and pH*0 are the parameters of the third-order model (with values −0.0235, 0.325,−1.1563, and 1.2262, respectively). And *pH* represents the pH of the soil studied.

Therefore, the Si(OH)_4_ available (SiAv) to be absorbed by the tomato plant is described by the following equation:

(6) $$\begin{array}{r} \text{SiAv}=\left(SiP-SiWater\right)*ETem*EOM*EpH+SiWater \end{array}$$

Where *SiP* is the maximum amount of silicon in a soil without polymerization [192.18 mg L^−1^ Si(OH)_4_]; *ETem* represents the effect of temperature on the availability of silicon (Equation 3); *EOM* describes the impact of organic matter on the availability of silicon (Equation 4); *EpH* represents the effect of pH on the availability of silicon (Equation 5); and *SiWater* is the amount of Si(OH)_4_ available in the irrigation water. The model supposes that under no condition will be the available Si(OH)_4_ be higher than the SiP value.

The accumulated Si (as SiO_2_) in the tomato plant (SiT) was determined with the silicon [Si(OH)_4_] available and the transpiration (Equation 1) in the following way:

(7) $$\begin{array}{r} \text{SiT}=\text{SiAv}*Transpiration*SiSi \end{array}$$

Where *SiSi* is the fraction of cumulative Si in the plant of the total available Si(OH)_4_ (g).

In soilless cultivation conditions, the only source of Si(OH)_4_ for the crop will be the content of the irrigation water since there is no such source of soil mineral replacement as in the soil. Therefore, the accumulation of Si in the tomato plant in soilless culture (ASiTSC) will depend entirely on the transpiration of the plant (Equation 1) and the availability of Si(OH)_4_ in the irrigation water (SiWater). This relationship is expressed as:

(8) $$\begin{array}{r} \text{ASiTSC}=Transpiration*SiWater*SiSi \end{array}$$

As previously described, the availability of silicon in soil depends on three primary conditions: pH, organic matter, and soil temperature. Of these conditions, it is feasible to modify the amount of organic matter or the pH. In the case of temperature, the easiest way would be to use covers as plastic mulches, which could increase the soil temperature by 3–4°C (Ruíz-Machuca et al., 2015). Therefore, these factors can be considered as crucial factors to the agronomic management of silicon availability (Liang et al., 2015).

### Silicon accumulation in tomato

**Figure 1:**
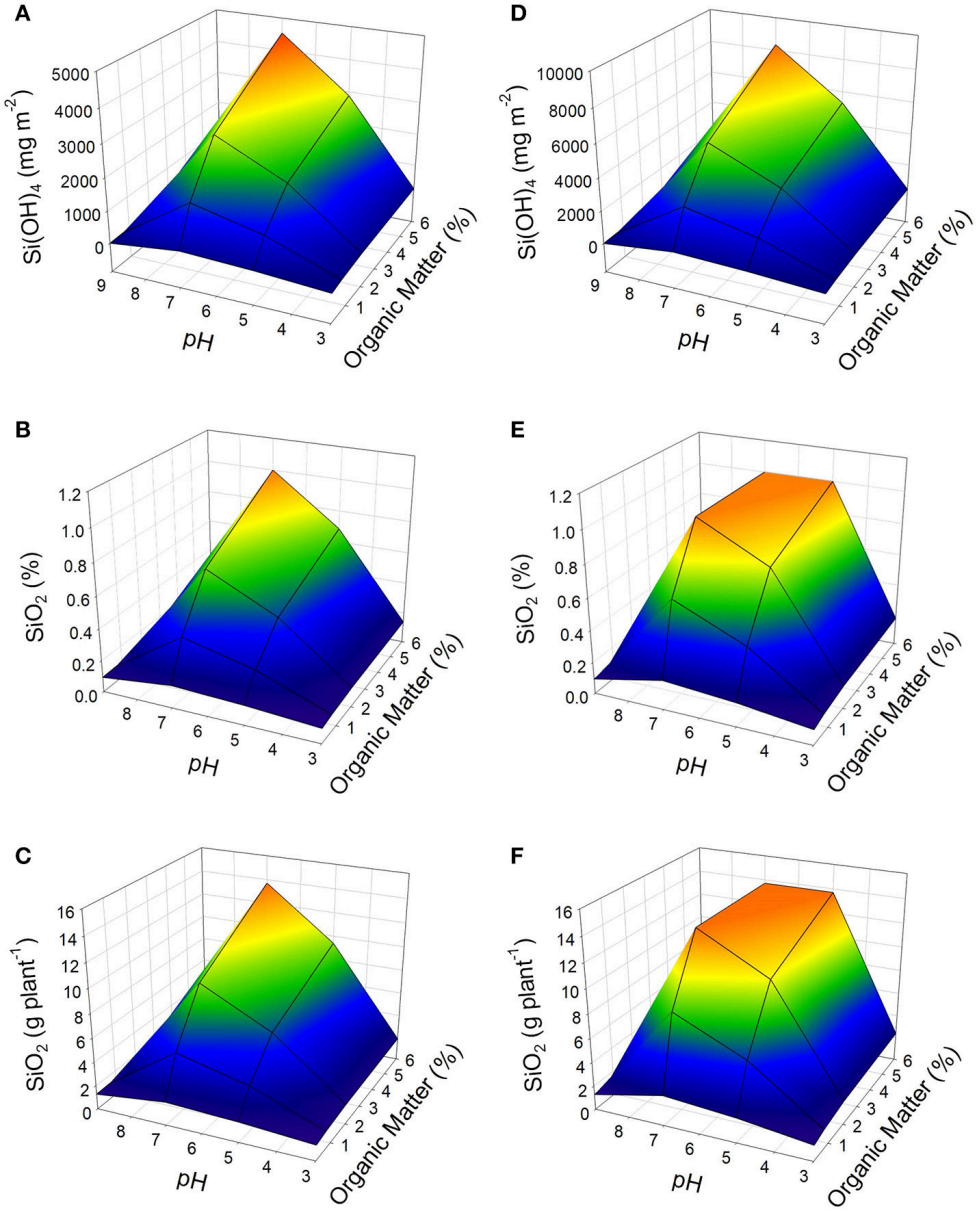
Effect of pH and content of organic matter on the availability of Si(OH)_4_ in soil **(A,D)**, concentration of Si (as SiO_2_) in plant dry weight **(B,E)**, and its accumulation in the tomato plant **(C,F)** under two conditions of average soil temperature: 15°C **(A–C)** and 25 ° C **(D–F)**. Data obtained from the proposed dynamic model considering as inputs of the growth model: PAR = 400 μmol m^−2^ s^−1^ (12 h per day); CO_2_ = 400 ppm; and Temperature: 30°C. As soil conditions were considered: a soil profile of 30 cm, soil humidity of 25% (w/w), and availability of Si as Si(OH)_4_ in irrigation water of 5 mg L^−1^.

According to the simulations carried out using the proposed model, a soil with pH 7.0 and organic matter content of 6% can obtain the maximum availability of Si(OH)_4_, which can be > 4,500 mg m^−2^ at 15°C; or > 8,300 mg m^−2^ at 25°C (Figures 1A,D). Considering the availability, and two average temperature conditions of soil (15 and 25°C), the highest availability of silicon is obtained with an average soil temperature of 25°C (Figure 1D). On the contrary, when the organic matter content is low (<1%) along with a pH >8 (like a soil of a semi-arid region), the availability of Si(OH)_4_ in the soil can drastically decrease to <20 mg m^−2^ at 15°C (Figure 1A), or <34 mg m^−2^ at 25° C (Figure 1D). These results describe the effect of pH, organic matter, and temperature factors on the availability of Si(OH)_4_ in soil. In addition to demonstrating the potential sensitivity of the availability of SiOH_4_ in the soil to the modifications on any of the conditions mentioned.

The availability of silicon in the soil will directly impact the accumulation of plants grown on it (Epstein, 2001). The higher availability of silicon derived from the factors evaluated (Figure 1D), results in a more significant accumulation of Si (as SiO_2_) based on the dry weight of the tomato plant (up to 1 %), as can be seen in Figure 1E. The Figure 1E fits well the reported Si concentration in tomato plants grown under pH 5.5 (Miyake and Takahashi, 1978) to pH 8.48 (Gunes et al., 2007). The same behavior is observed concerning accumulated silicon per plant, reaching up to 13.7 g per plant, that represent the maximum accumulation of silicon for tomato plants under this conditions (Figure 1F). However, low availability of silicon in the soil can lead to a small accumulation of silicon in the plant. In the example the conditions of a soil corresponding to the situation of a semi-arid region, there would be an accumulation <1.3 g per plant for both soil temperature conditions (Figures 1C,F). This equals to a silicon concentration based on plant dry weight <0.1% (Figures 1B,E).

Since silicon accumulates in the different organs of the tomato plant as a function of the rate of transpiration, then the highest accumulation will be observed in the leaves, since they represent 90% of total transpiration. In fruits, a lower accumulation of silicon will be seen, since the rate of transpiration is little compared to that of the leaves (~5%) (Leonardi et al., 2000). However, the availability of silicon in the soil will ultimately define the accumulation of silicon both in the entire plant and in its various organs.

Contrary to a crop established in soil, in soilless cultivation, e.g., hydroponics, the primary factor that will modify the availability of Si for the plant will be its concentration in the irrigation water used. It has been reported that irrigation water can have a Si content [as Si(OH)_4_] of 5–20 mg L^−1^, while it is considered that an adequate concentration of Si would be 28 mg L^−1^ (Epstein, 1994, 1999; Liang et al., 2015). However, the transpiration rate of the crop will finally define the amount of SiOH_4_ absorbed and accumulated in the different organs. Since the growth of the plant and the proper distribution of biomass in the various organs will affect the rate of transpiration, then crop growth should be considered as an additional factor that will change the accumulation of silicon in a soilless crop system. Therefore, the environmental factors (PAR, CO_2_, air temperature) that affect the growth of the crop will, in turn, affect the accumulation of silicon in the different organs.

**Figure 2:**
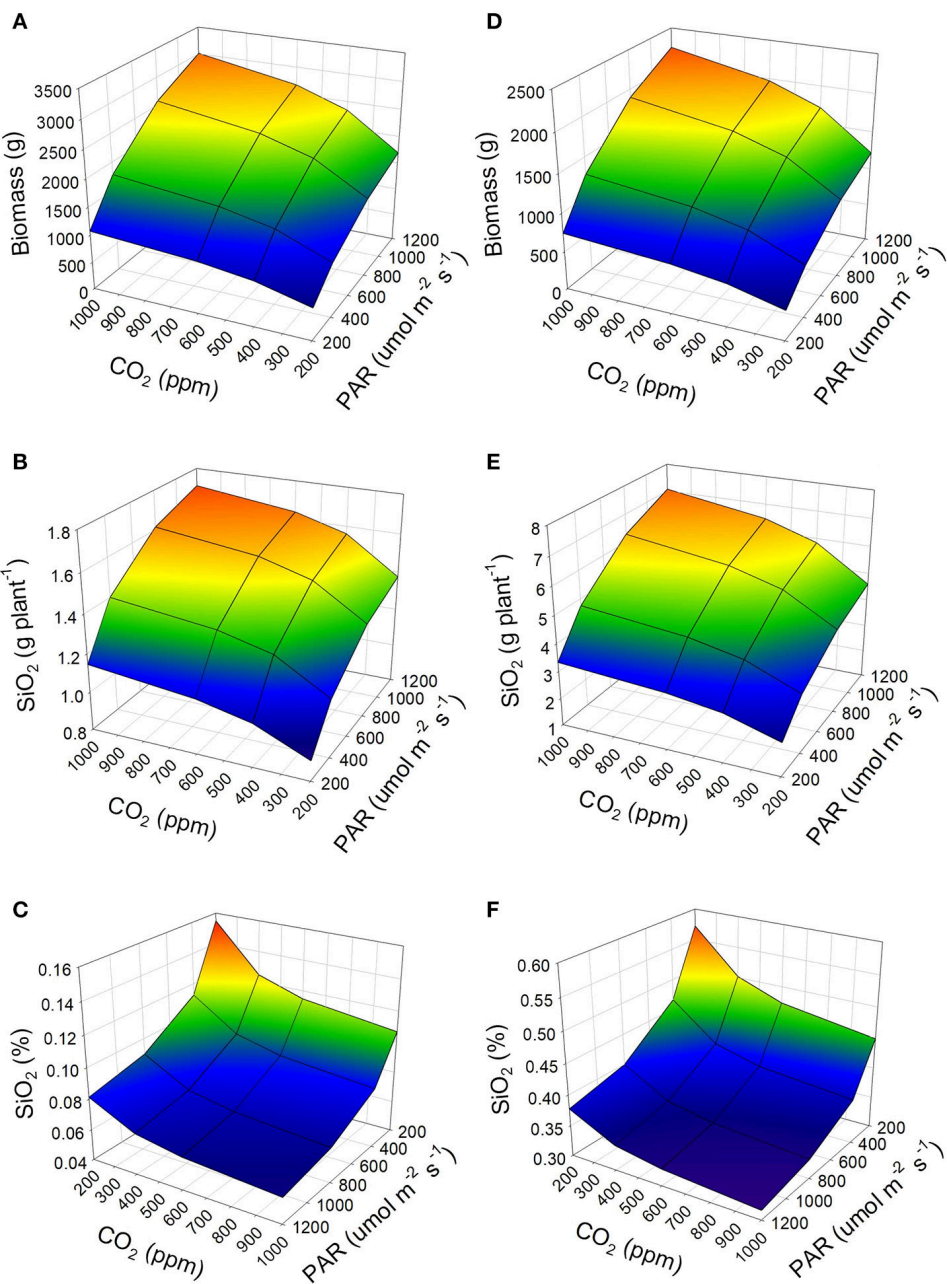
Accumulation of dry biomass of the tomato plant under two conditions of air temperature 30°C **(A–C)** and 20°C **(D–F)**, and its impact on the accumulation **(B,E)** and concentration **(C,F)** of silicon (as SiO_2_) in the plant. It is considered a tomato crop developed in a soilless cultivation system, an availability of Si as Si(OH)_4_ in the irrigation water of 5 mg L^−1^, and 12 h of PAR per day.

According to the simulations carried out, a low concentration of CO_2_ and a low incidence of PAR generate little accumulation of biomass in the tomato plant (Figures 2A,D). This same result is observed when the temperature of the environment changes, 30°C generates biomass of up to 3,000 g per plant (Figure 2A); while 20°C produces up to 2,240 g per plant (Figure 2D), at the highest conditions of PAR and CO_2_ concentration. As a consequence, the total transpiration of the plant is modified when environmental factors changes, and therefore the accumulation of silicon. With an average temperature of 30°C, the biomass accumulated in the fruits can represent around 60% of the total of the tomato plant, and the leaves less than 10%. However, when the temperature drops to 20°C, the biomass distribution in the tomato plant changes. Under this condition, the biomass accumulated in the fruits is ~33%, whereas in the leaves it increases up to 33%.

Since the leaves constitute the largest area of transpiration, modifying the temperature of the environment in which the tomato grows substantially alters the final accumulation of silicon. Therefore, according to the model, a temperature of 30°C will lead to a lower accumulation of silicon in the tomato plant (Figure 2B), while at 20°C there will be more significant accumulation (Figure 2E). The Figure 2C fits the reported Si concentration in tomato plants grown under temperatures of 28–32°C (Cao et al., 2015). The result will be a higher level of silicon in dry weight of the tomato plant at low temperatures (Figure 2F), while high temperatures will decrease the concentration considerably (Figure 2C).

**Figure 3:**
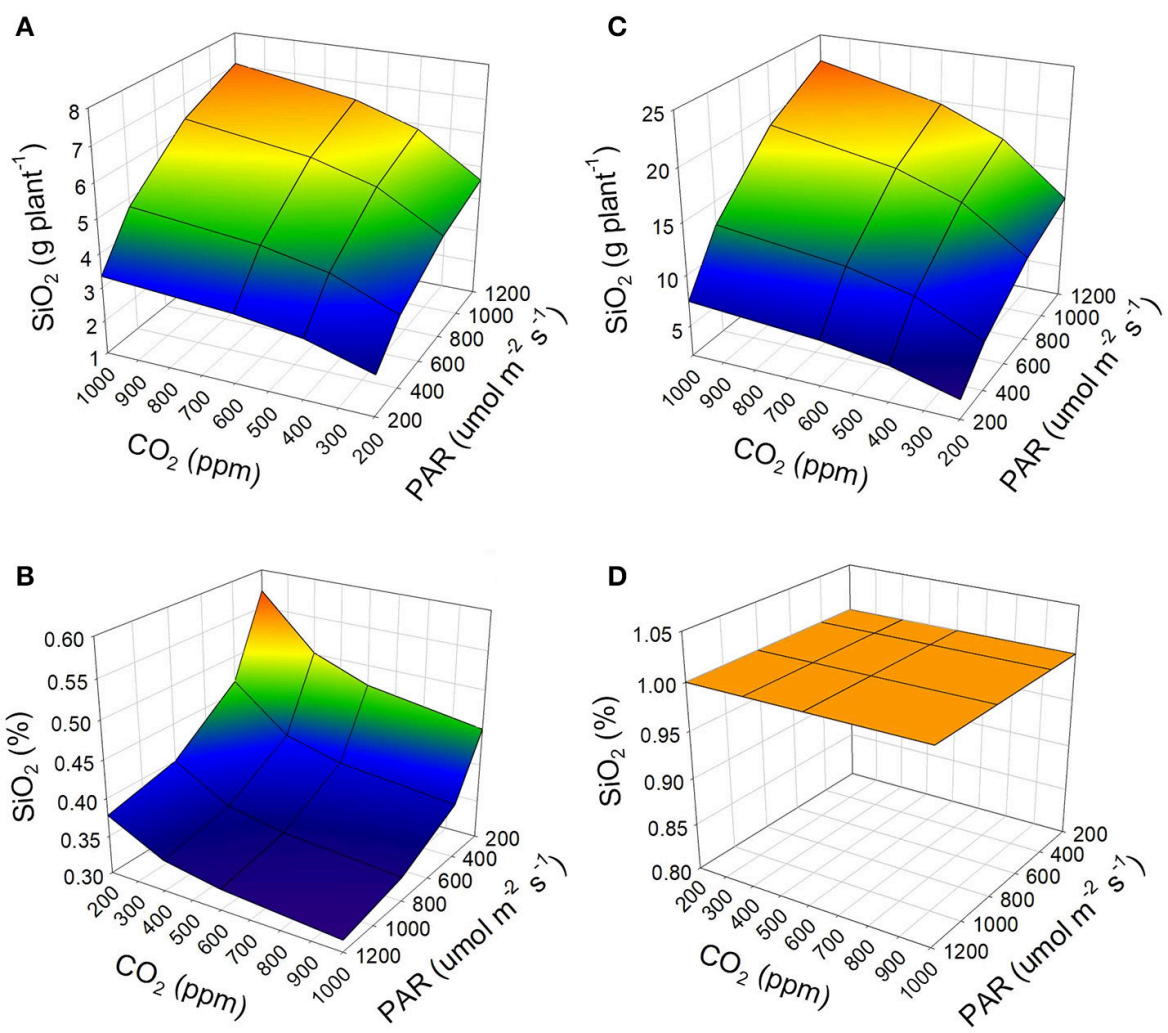
Effect of the availability of Si as Si(OH)_4_ in the irrigation water on the accumulation **(A,C)** and concentration **(B,D)** of silicon (as SiO_2_) in the tomato plant developed under a soilless cultivation system. 5 mg L^−1^ of Si(OH)_4_ **(A,B)** represent low availability of Si and 28 mg L^−1^ of Si(OH)_4_ represent adequate availability **(C,D)**. For the simulation, 12 h of PAR and an average air temperature of 20°C were considered.

When considering the availability of silicon in the irrigation water, it can be observed that a condition of low Si content (5 mg L^−1^ as Si[OH]_4_) will result in less concentration and accumulation of silicon in the plant (Figures 3A,B). On the contrary, adequate availability of Si (28 mg L^−1^ as Si[OH]_4_) in the irrigation water will result in increased accumulation and concentration of silicon in the plants (Figures 3C,D). High accumulation appears to occur regardless of the environmental conditions in which the crop develops, when there is adequate availability of Si in the irrigation water. However, when conditions are favorable for the development of leaves in tomato plants, the maximum concentration of silicon for this species can be reached (Figure 3D).

The model presented focuses on the impact of external factors on the growth of tomato plants, under the assumption that as long as exists the availability of Si the plants absorb it and transport it at a rate proportional to the growth rate and transpiration rate. The point highlighted with the model is that the biofortification of the fruits with Si depends on the availability of the element in the form of Si(OH)_4_ both in soil and in soilless culture.

Sakurai et al. (2017) developed a successful dynamic model of Si absorption and transport for rice. The model is based on endogenous variables, as the activity of different transporters and the distribution of Si through different nodes in the entire plant. The model presented in this manuscript is focused on exogenous variables, susceptible to agronomic management both in field cultivation as in the greenhouse, and it has been used successfully to simulate the absorption of other elements in tomato and other crops (Juárez-Maldonado et al., 2014b; Ramírez-Pérez et al., 2018).

However, it must be taken into account that the presented model is used to describe the accumulation of silicon in the plant under relatively favorable environmental situations. The presence of stresses such as water deficit, salinity, deficiency of mineral nutrients and pathogens, results in loss of precision. With a certain amount of PAR and with a particular temperature regime, the stressed plants would have real biomass lower than the estimated by the model, which means an overestimation of the absorbed silicon.

As far as we know, except those published by (Sakurai et al., 2015, 2017) for monocotyledons, there are no similar dynamic models published about the absorption of silicon in dicots. The model described for the tomato crop is a first preliminar advance that we believe substantially can improve the understanding of some factors that regulate the bioavailability of silicon.

## Conclusions

After the results obtained from the presented model, the following is proposed:

1. When crops are grown in soil, the bioavailability of silicon can be increased by adding organic matter from organic amendments or humic substances, or by modifying the pH of the soil solution, using organic or inorganic acids, to be the closest to 7.0.
2. In soilless crop systems, the best is to increase the Si content in the irrigation water. Preferably to have at least 28 mg L^−1^ of Si, equivalent to 96.1 mg L^−1^ of Si(OH)_4_.
3. The majority of the soils used for agriculture in tropical or subtropical semiarid or arid areas have conditions that do not favor the availability of Si(OH)_4_, or the irrigation water has a low concentration of silicon. Therefore, it is advisable to apply silicates of sodium, potassium, or calcium as part of the fertilization program to favor the accumulation in the plants. In the long term, the availability of Si can be increased in the soil using mineral sources rich in Si, such as siliceous sands.

## Author contributions

All authors were responsible for processing information and manuscript writing. AB-M, AJ-M, FP-L, and AM-D: Conceptualization; AJ-M, ML-P, LR-P, and AM-D: Model design and implementation; ML-P, FP-L, SG-M, LG-D, JG-M, and AB-M: Manuscript drafting. All authors read and approved the final manuscript.

### Conflict of interest statement

The authors declare that the research was conducted in the absence of any commercial or financial relationships that could be construed as a potential conflict of interest.
